# Kyste arachnoidien intra et supra sellaire: apport de l'IRM

**DOI:** 10.11604/pamj.2015.22.245.7535

**Published:** 2015-11-13

**Authors:** Mohamed Badaoui, Soufiane Belabbes, Abdellatif Darbi, Abdennasser El Kharras

**Affiliations:** 1 Service de Medecine Interne, 1er Centre Médico-chirurgical, Agadir, Maroc; 2 Service d'Imagerie Médicale. 1^er^ Centre médico-chirurgical, Agadir, Maroc; 3 Service d'Imagerie Médicale, Hospital Militaire Mohamed V, Rabat, Maroc

**Keywords:** Kyste arachnoidien, selle turcique, IRM hypophysaire, arachnoid cyst, pituitary fossa, pituitary MRI

## Abstract

Les kystes arachnoïdiens intrasellaires sont rares et souvent étendus en suprasellaire. Leur diagnostic doit être évoqué devant toute formation de nature kystique dont le signal est identique à celui du liquide cérébro-spinal. Nous rapportons le cas d'un kyste arachnoïdien de siège intra et suprasellaire révélé par une baisse de l'acuité visuelle et une hémianopsie bitemporale dont l'exploration par l'IRM cérébrale retrouve la formation kystique de signal liquidien identique à celui du LCS, avec une prise de contraste localisée de la paroi postéro-latérale gauche du kyste qui correspond à la glande hypophysaire déplacée et étalée. Un abord chirurgical par voie trans-sphénoïdale a permis d’évacuer le contenu kystique et de décomprimer les voies optiques. L’évolution a été marquée par une amélioration immédiate des perturbations visuelles.

## Introduction

Les kystes arachnoïdiens intra sellaire sont extrêmement rares. Leur siège habituel est la fosse cérébrale médiane. C'est dans ce sens de leur rareté que nous rapportons ce nouveau cas exploré par l'IRM.

## Patient et observation

**Figure 1:**
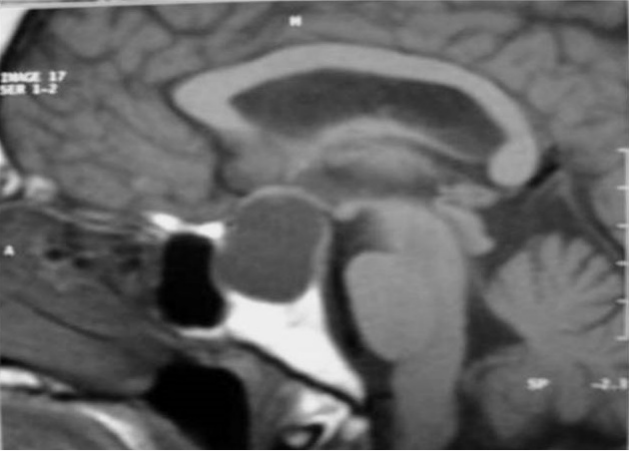
IRM en coupe sagittale T1. Elargissement de la cavité sellaire par une lésion expansive intra et suprasellaire se présentant en hyposignal

**Figure 2:**
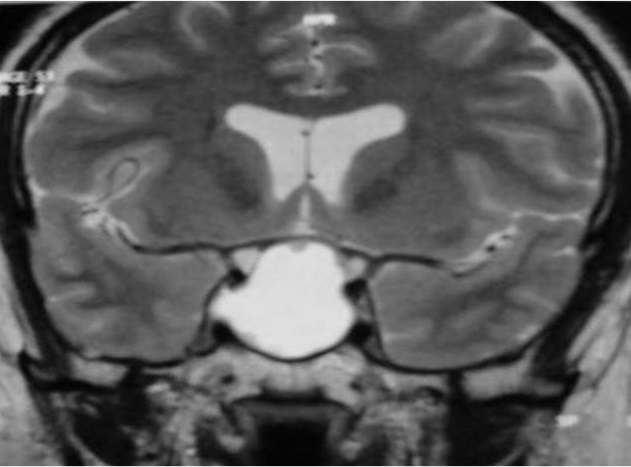
IRM en coupe frontale T2. Hypersignal franc du contenu kystique identique à celui du LCS

**Figure 3:**
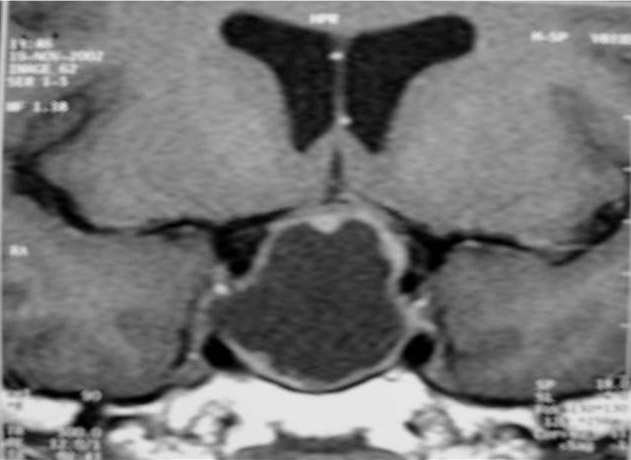
IRM en coupe frontale avec injection de gadolinium. Ecrasement du chiasma optique et prise de contraste postéro latérale gauche de la paroi kystique correspondant à l'hypophyse normale déplacée

**Figure 4:**
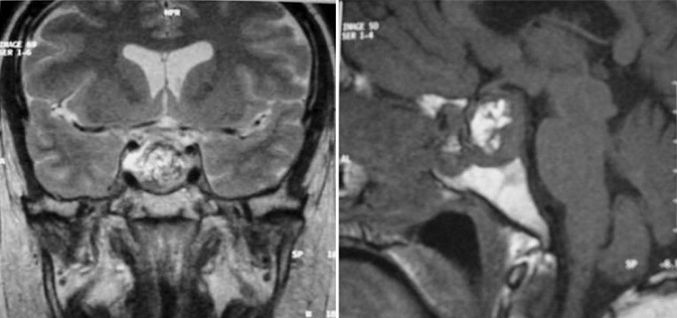
IRM de contrôle en post opératoire. Affaissement du kyste et décompression du chiasma optique

Mr.B.B. est un patient âgé de 32 ans, sans antécédent pathologique notable, qui accusait depuis 2 mois des troubles visuels associés à des céphalées surtout frontales sans signes endocriniens associés. A l'admission le patient était conscient; sans déficit neurologique, mais présentant: une amputation des deux hemi-champs temporaux, une baisse de l'acuité visuelle bilatérale prédominante à gauche, une pâleur papillaire de l'oeil gauche et une atrophie de la partie temporale de la papille droite au fond d'oeil. Le reste de l'examen somatique était normal. Une IRM est réalisée selon des coupes multi planaires avec des séquences SE pondérées T1 et T2 avant et après injection de gadolinium a objectivé un important processus expansif, de siège intra sellaire avec extension supra sellaire, bien limité, de signal liquidien en hyposignal franc sur les séquences en SET1 (Figure 1) et hypersignal intense en SET2 (Figure 2) identique à celui du LCS, sans rehaussement de sa paroi après injection de gadolinium (Figure 3). Ce processus refoule vers le haut en l’écrasant le chiasma optique. Le champ visuel a confirmé l'existence de l'hémianopsie bitemporale. Le bilan endocrinien était sans anomalie. La chirurgie par voie trans-sphénoïdale a permis l’évacuation du kyste contenant un liquide semblable au LCS et une exérèse partielle de la paroi kystique. La selle turcique a été comblée par du tissu graisseux pour prévenir une selle turcique vide. L’évolution a été favorable. L'IRM de contrôle a montré l'affaissement du kyste avec décompression des voies optiques (Figure 4).

## Discussion

Décrit pour la première fois en 1831 par BRIGHT, le kyste arachnoïdien est une formation, dont la paroi est formée par l'arachnoïde, contenant du liquide cérébro-spinal sous tension. Les kystes arachnoïdiens intrasellaires sont très rares, ils représentent moins de 10% des cas [1]. Deux théories essaient d'expliquer leur pathogénie. La première par l’évolution de selles turciques vides, et la deuxième par duplication de la membrane arachnoïdienne située sous le diaphragme sellaire [2]. Cliniquement, l'hypertension intracrânienne, la symptomatologie endocrinienne par compression hypothalamo-hypophysaire de type plutôt déficitaire, et la symptomatologie visuelle par compression du chiasma et des voies optique sont les principaux motifs de consultation [1, 3].

L'IRM reste le moyen le plus efficace pour faire le diagnostic. Ce dernier reste tout de même relativement délicat. En effet, les diagnostics différentiels du kyste arachnoïdien intrasellaire sont nombreux, Ils sont représentés essentiellement par l'adénome kystique, kyste de Rathke, craniopharyngiome kystique, kyste épidermoide, abcès hypophysaire et kyste parasitaires [2, 3]. Les éléments utiles pour le diagnostic différentiel sont représentés par le signal du contenu du kyste et par l'absence ou la présence d'une prise de contraste au niveau de la paroi du kyste [4]. En Effet, les adénomes kystiques, les kystes de la poche de Rathke et les craniopharyngiomes kystiques, présentent le plus souvent un contenu dont le signal est différent de celui du liquide cérébro-spinal en raison d'un contenu protidique élevé. C'est essentiellement en IRM en densité protonique et en T2 que cette différence de signal apparaît [2, 5]. Dans certains cas, le contenu peut déjà apparaître hyper intense en T1. En plus du signal du contenu, la paroi des autres formations est le plus souvent épaissie, irrégulière avec un rehaussement lors de l'injection du produit paramagnétique. La prise de contraste localisée du kyste n’élimine pas le diagnostic, car il peut s'agir d'un rehaussement normal d'une hypophyse déplacée et étalée sur la paroi du kyste [4].

Il est important de faire le diagnostic en préopératoire du kyste arachnoïdien, car la chirurgie trans-sphénoïdale n'est pas recommandée dans ce type de lésions vues la fréquence élevée des complications post opératoires (rhinorrhées, troubles visuels, méningites et abcès de la cavité sellaire) qui est de l'ordre de 28% alors qu'elles n'est que de 2,3% dans la chirurgie trans-sphénoïdale des adénomes [4, 5]. C'est pourquoi, certains auteurs recommandent une approche sous frontale de ces kystes pour éviter ces complications [5].

## Conclusion

Les kystes arachnoïdiens intrasellaires sont rares. De ce fait, leur diagnostic est difficile. L'IRM est le moyen d'imagerie qui permet de suspecter fortement le diagnostic. Toute suspicion de kyste arachnoïdien intrasellaire doit faire préférer une approche chirurgicale sous frontale plutôt que transrhinoseptale.
